# Assessment of the Diagnostic Accuracy of Artificial Intelligence Software in Identifying Common Periodontal and Restorative Dental Conditions (Marginal Bone Loss, Periapical Lesion, Crown, Restoration, Dental Caries) in Intraoral Periapical Radiographs

**DOI:** 10.3390/diagnostics15111432

**Published:** 2025-06-04

**Authors:** Wael I. Ibraheem, Saurabh Jain, Mohammed Naji Ayoub, Mohammed Ahmed Namazi, Amjad Ismail Alfaqih, Aparna Aggarwal, Abdullah A. Meshni, Ammar Almarghlani, Abdulkareem Abdullah Alhumaidan

**Affiliations:** 1 Department of Preventive Dental Sciences, College of Dentistry, Jazan University, Jazan 45142, Saudi Arabia; wibraheem@jazanu.edu.sa; 2 Department of Prosthetic Dental Sciences, College of Dentistry, Jazan University, Jazan 45142, Saudi Arabia; ameshni@jazanu.edu.sa; 3 Intern Clinic, College of Dentistry, Jazan University, Jazan 45142, Saudi Arabia; dr.moha.ayoub@gmail.com (M.N.A.); mohammed.namazi@hotmail.com (M.A.N.); amjadalfaqih00@gmail.com (A.I.A.); 4 Vitaldent Advanced Dental Clinic, Faridabad 121002, India; draparna1980@gmail.com; 5 Department of Periodontics, Faculty of Dentistry, King Abdulaziz University, Jeddah 21589, Saudi Arabia; ammar.almarghlani@gmail.com; 6 Department of Preventive Dental Sciences, College of Dentistry, Imam Abdulrahman Bin Faisal University, Dammam 31441, Saudi Arabia; aaalhumaidan@iau.edu.sa

**Keywords:** artificial intelligence, diagnostic imaging, diagnosis, digital imaging/radiology, convolutional neural network (CNN), machine learning, deep learning, intraoral radiographs, marginal bone loss, dental caries, periapical lesion, dental crown, calculus, restoration

## Abstract

**Objectives:** The purpose of the study is to evaluate the diagnostic accuracy of artificial intelligence (AI) software in detecting a common set of periodontal and restorative conditions, including marginal bone loss, dental caries, periapical lesions, calculus, endodontic treatment, crowns, restorations, and open crown margins, using intraoral periapical radiographs. Additionally, the study will assess how this AI software influences the diagnostic accuracy of dentists with varying levels of experience in identifying these conditions. **Methods:** A total of three hundred digital IOPARs representing 1030 teeth were selected based on predetermined selection criteria. The parameters assessed included (a) calculus, (b) periapical radiolucency, (c) caries, (d) marginal bone loss, (e) type of restorative (filling) material, (f) type of crown retainer material, and (g) detection of open crown margins. Two oral radiologists performed the initial diagnosis of the selected radiographs and independently labeled all the predefined parameters for the provided IOPARs under standardized conditions. This data served as reference data. A pre-trained AI-based computer-aided detection (“CADe”) software (Second Opinion^®^, version 1.1) was used for the detection of the predefined features. The reports generated by the AI software were compared with the reference data to evaluate the diagnostic accuracy of the AI software. In the second phase of the study, thirty dental interns and thirty dental specialists were randomly selected. Each participant was randomly assigned five IOPARs and was asked to detect and diagnose the predefined conditions. Subsequently, all the participants were requested to reassess the IOPARs, this time with the assistance of the AI software. All the data was recorded using a self-designed Performa. **Results:** The sensitivity of the AI software in detecting caries, periapical lesions, crowns, open crown margins, restoration, endodontic treatment, calculus, and marginal bone loss was 91.0%, 86.6%, 97.1%, 82.6%, 89.3%, 93.4%, 80.2%, and 91.1%, respectively. The specificity of the AI software in detected caries, periapical lesions, crowns, open crown margins, restoration, endodontic treatment, calculus, and marginal bone loss was 87%, 98.3%, 99.6%, 91.9%, 96.4%, 99.3%, 97.8%, and 93.1%, respectively. The differences between the AI software and radiologist diagnoses of caries, periapical lesions, crowns, open crown margins, restoration, endodontic treatment, calculus, and marginal bone loss were statistically significant (all *p* values < 0.0001). The results showed that the diagnostic accuracy of operators (interns and specialists) with AI software revealed higher accuracy, sensitivity, and specificity in detecting caries, PA lesions, restoration, endodontic treatment, calculus, and marginal bone loss compared to that without using AI software. There were variations in the improvements in the diagnostic accuracy of interns and dental specialists. **Conclusions:** Within the limitations of the study, it can be concluded that the tested AI software has high accuracy in detecting the tested dental conditions in IOPARs. The use of AI software enhanced the diagnostic capabilities of dental operators. The present study used AI software to detect a clinically useful set of periodontal and restorative conditions, which can help dental operators in fast and accurate diagnosis and provide high-quality treatment to their patients.

## 1. Introduction

Digitalization in dentistry has transformed how dental professionals deliver care to their patients. The integration of new technologies has significantly surpassed traditional methods of managing dental practices. Tools such as digital scanners [1], CAD/CAM systems [2], and robotics aid in providing high-quality treatments to their patients [3].

Artificial intelligence (AI) is being considered as the next industrial revolution and has improved overall productivity in every field. AI has three major components: domain knowledge, data generation, and machine learning [4,5]. The plethora of applications that AI can offer is still being explored and exceeds our current imagination. Since 2015, AI has been making strides in the medical field, particularly when used in conjunction with deep learning convolutional neural networks (CNNs) [5,6]. The goal of this exploration and development is to reduce reliance on human intelligence, or rather, to assist it. Early detection can lead to improved outcomes by avoiding invasive treatments, making procedures more cost effective, and increasing the accuracy of available systems.

A popular area within machine learning is “deep learning,” which utilizes multi-layered (deep) neural networks to extract hierarchical features from data [7,8]. For complex cases, such as image analysis, convolutional neural networks (CNNs) are commonly used to identify features like edges, corners, shapes, and larger patterns. Deep learning involves repeatedly passing data (e.g., images) and their corresponding labels (e.g., “carious tooth” or “specific area on an image indicating a caries lesion”) through the neural network during training. This process adjusts the model parameters, known as weights, iteratively to enhance the model’s accuracy [9,10]. In dentistry, AI has been utilized for various tasks, including dental image analysis for detecting dental caries [11,12,13,14,15,16], identifying landmarks [17,18], classifying teeth [19,20], and segmenting restorations in photographs, radiographs, or surface scans [21]. It has also been used for pathology detection in radiographic, transillumination, or photographic images [22]. The interpretation of radiographic images has crucial applications. While AI-based software plays a significant role in general dental diagnostics, it has also been highlighted as an important advancement in detecting and classifying dental implants—a task that can be challenging even for practitioners. Several studies have addressed this issue using panoramic and periapical radiographs [23,24]. Additionally, research on 3D imaging has evaluated the performance of various CNNs for automatically detecting anatomical and dental structures, including the mandible, mandibular nerve canal, and pharyngeal airway [25,26].

While many AI applications in dentistry have emerged since 2019, there remains limited supporting data for their use in clinical settings. Studies have shown that AI demonstrates diagnostic accuracies that surpass those of individual dentists in various diagnostic accuracy studies [11,12]. Despite a range of studies evaluating the diagnostic performance of AI models, the continuous advancements in AI technology have motivated researchers to reassess these tools and enhance their understanding of them.

Currently, there are few published studies that evaluate the diagnostic accuracy of AI models specifically using periapical radiographs to identify periodontal and restorative dental conditions such as caries, bone loss, dental restorations, crowns, and periapical lesions [11,12,13,14,15,16,27,28,29,30,31]. To address this knowledge gap, the present study aims to evaluate the diagnostic accuracy of AI software in detecting a common set of periodontal and restorative conditions, including dental caries, marginal bone loss, periapical lesions, calculus, endodontic treatment, crowns, restorations, and open crown margins, using intraoral periapical radiographs. Additionally, the study will assess how AI software influences the diagnostic accuracy of dentists with varying levels of experience in identifying these conditions.

The parameters assessed for identification included marginal bone loss, dental caries, periapical lesions, crowns, crown margins, restorations, endodontic treatment, and calculus. The null hypothesis being examined posits two key points. (1) There will be no significant difference between the detection accuracy of the trained oral radiologist (reference data) and the AI-based software for the tested parameters in periapical radiographs. (2) The use of AI software will not influence the diagnostic accuracy of dental operators.

## 2. Materials and Methods

The current study assessed the performance of pre-trained AI software alongside dentists in identifying various findings in intraoral radiographs. The present study is a retrospective study with convenient sampling. To report the results, the study adhered to the protocols established by the STARD (Standards for Reporting of Diagnostic Accuracy Studies) and the CLAIM (Checklist for Artificial Intelligence in Medical Imaging) guidelines [32,33,34].

### 2.1. Image Dataset Preparation

The study was carried out at the College of Dentistry, Jazan University, following ethical approval from the Standing Committee for Scientific Research—Jazan University (Reference No. REC-45/05/887). Periapical radiographs of patients visiting the dental college were collected from the Electronic Health Record system (CS R4 Clinical, v6.1.1, Carestream Dental Ltd., Hertfordshire, UK). Two independent researchers randomly searched the patient database from June 2022 to November 2024.

Inclusion criteria consisted of intraoral periapical radiographs of patients over 18 years of age that were clear and displayed the full anatomical crown and root apex. Exclusion criteria included radiographs of deciduous teeth or mixed dentition, those with severe noise or haziness, radiographs of partially edentulous areas, teeth exhibiting abnormal crown or root anatomy, and any radiographs that were difficult to assess. All radiographs were captured by trained radiology technicians using standard settings on radiographic machines from Sirona (Sirona Dental systems GmbH, Bensheim, Germany).

To determine the sample size for this study, earlier published research was consulted [11,12]. Subsequently, G*Power software (version 3.1.9.7, 2020; Heinrich Heine University, Düsseldorf, Germany) was employed to confirm the sample size. A study power of 80% was maintained to detect a difference with an effect size (f) of 0.40 and an alpha error value of 5%. This analysis indicated that a minimum of 980 teeth needed to be assessed, assuming a limited correlation between the groups.

A total of 300 digital IOPARs, representing 1030 teeth, were selected. All the radiographs were acquired using the parameters recommended by the manufacturer, which include an X-ray tube voltage of 70 kV and an X-ray tube current of 7 mA. The exposure time varied from 0.25 to 0.64 s based on the patient’s physical type. Standard digital holders were used. There were no reference markers on the X-rays. A third researcher reviewed all the selected radiographs, which were exported in DCM format. The IOPARs were labeled, and all patient-related details were removed prior to their use with the AI diagnostic software. All the X-rays were blinded to examiners. Each tooth on the selected IOPA was labeled according to the FDI scheme by one researcher and subsequently rechecked by another researcher. The selection of IOPARs did not take into account any differences in sex or race. The periodontal and restorative conditions assessed included (a) marginal bone loss (b) periapical radiolucency, (c) caries, (d) calculus, (e) type of restorative (filling) material, (f) type of crown retainer material, and (g) detection of open crown margins.

Two oral radiologists, each with over five years of experience, were responsible for the initial diagnosis of the selected radiographs, which served as reference data. These two experts independently labeled all the predefined parameters for the provided IOPARs (200 each) under standardized conditions (standardized pixels and dimly lit rooms) [12]. The Cohen test of inter-observer reliability revealed a score of 0.95 between the two oral radiologists. A third radiologist reconfirmed all the labeled images and engaged in discussions with the previous two radiologists in case of any disagreements.

### 2.2. AI Software Architecture

Pre-trained computer-aided detection (“CADe”) software (Second Opinion^®^) version 1.1 (Pearl Inc., West Hollywood, CA, USA) was used in the present study to analyze all selected IOPARs. Second Opinion^®^ uses computer vision technology, developed using machine learning techniques, to detect and draw attention to regions on bitewing and periapical radiographs where pathologic and non-pathologic dental features may appear [35]. The software can be used on a standard chair-side PC.

This system has three main components: (a) an in-office application or user interface, (b) an Application Programming Interface (“API”), and (c) computer vision (CV). Once a new IOPAR image to be diagnosed is uploaded in the local folder by the dentist, the cloud-based APIs are invoked, which submit images to CV models for processing. The metadata produced by CV models detect and describe the type and location of the detected pathologic or non-pathologic features that may appear in the radiographs. This data is sent back to the user interface for visual display, where the detected features will appear within boundary boxes overlaid on the original radiograph. The software detects these features based on visual appearance that closely resembles the known features used during the training of the AI model. The average interference time per radiograph was approximately 1 min. The outcomes can be crosschecked by the dentist, who can also report mistakes, which helps further develop the software in case of a wrong or missing diagnosis [35].

**Figure 1:**
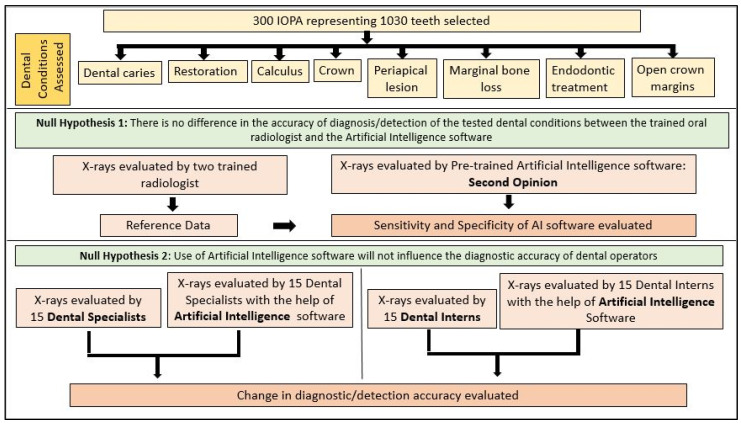
Flowchart explaining the methodology used in the study.

**Figure 2:**
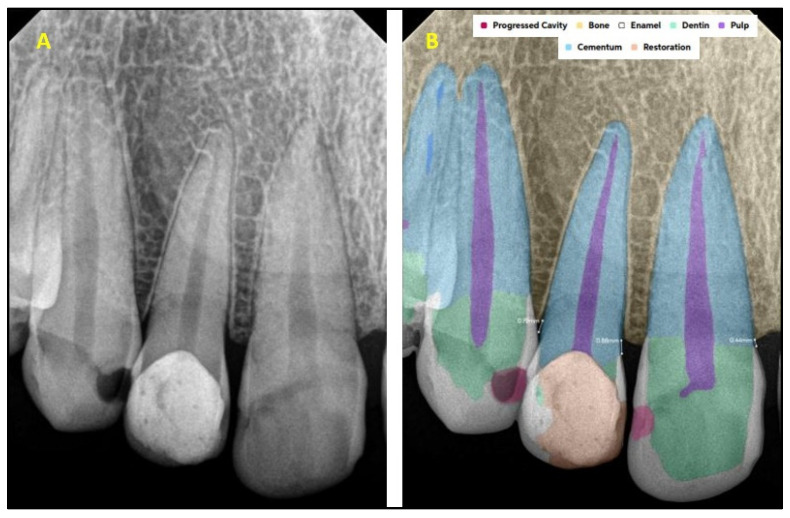
(**A**) Intraoral periapical radiographic image of maxillary anterior teeth; (**B**) analysis made by artificial intelligence software depicting diagnosed conditions (dental caries, restoration, bone loss).

**Figure 3:**
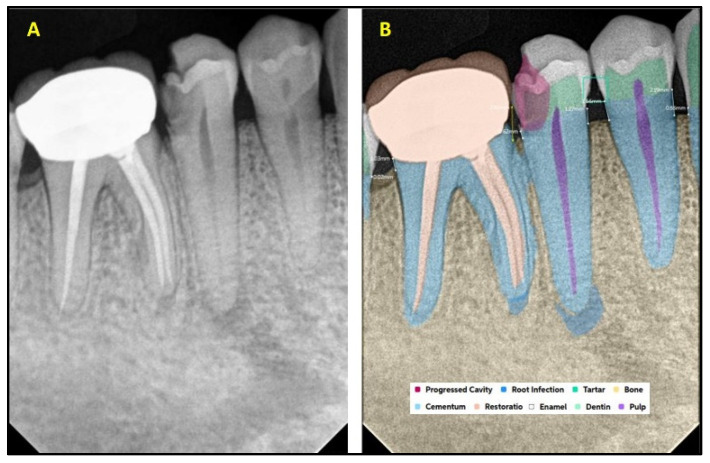
(**A**) Intraoral periapical radiographic image of mandibular posterior teeth; (**B**) analysis made by artificial intelligence software depicting diagnosed conditions (dental caries, crown, bone loss, periapical lesion, endodontic treatment, and calculus).

**Figure 4:**
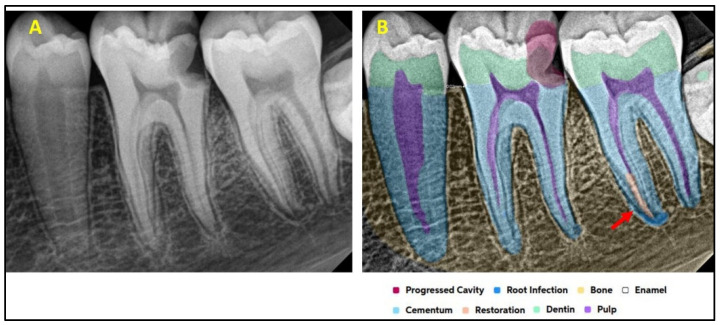
(**A**) Intraoral periapical radiographic image of mandibular posterior teeth; (**B**) analysis made by artificial intelligence software depicting inaccurately diagnosed condition: restoration in the root portion of the mandibular second molar (marked with a red arrow).

**Figure 5:**
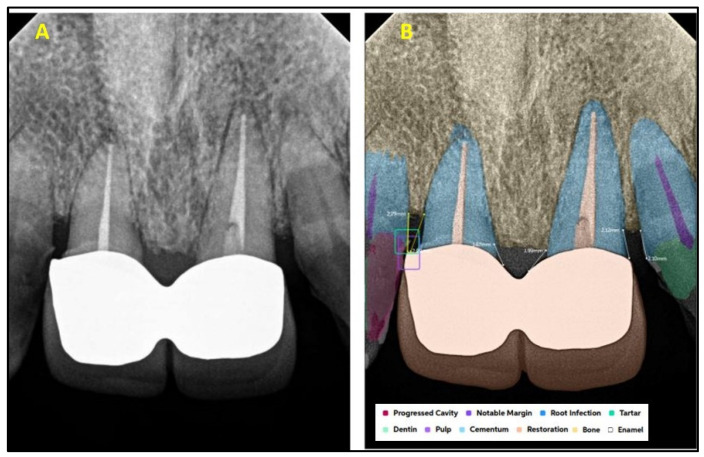
(**A**) Intraoral periapical radiographic image of maxillary anterior teeth; (**B**) analysis made by artificial intelligence software depicting diagnosed conditions (dental caries, crowns, open crown margins, bone loss, periapical lesion, endodontic treatment, and calculus).

**Figure 6:**
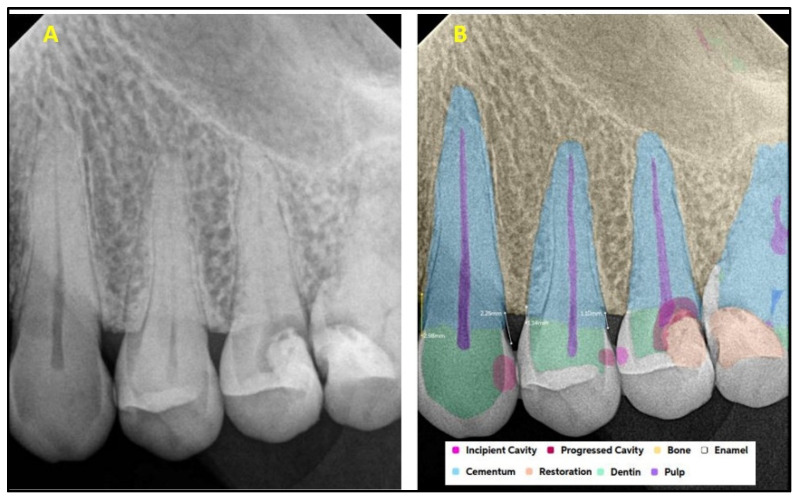
(**A**) Intraoral periapical radiographic image of maxillary posterior teeth; (**B**) analysis made by artificial intelligence software depicting diagnosed conditions (dental caries, restorations, and bone loss).

**Figure 7:**
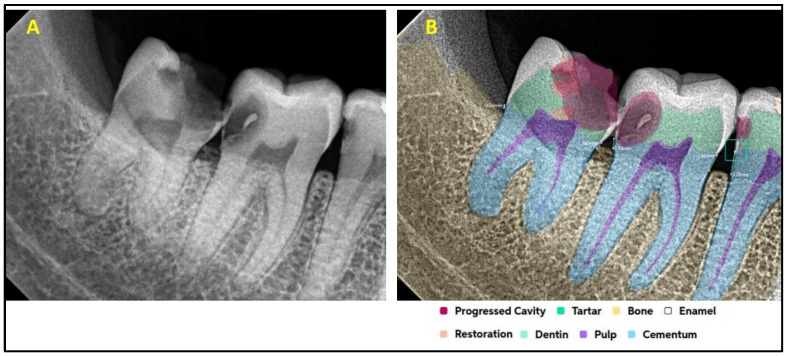
(**A**) Intraoral periapical radiographic image of mandibular posterior teeth; (**B**) analysis made by artificial intelligence software depicting diagnosed conditions (dental caries, bone loss, restoration, and calculus).

**Figure 8:**
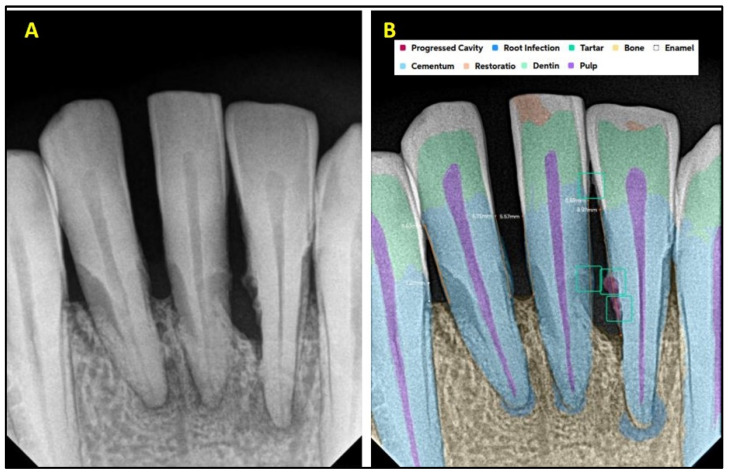
(**A**) Intraoral periapical radiographic image of mandibular anterior teeth; (**B**) analysis made by artificial intelligence software depicting diagnosed conditions (dental caries, bone loss, periapical lesion, restoration, and calculus).

**Figure 9:**
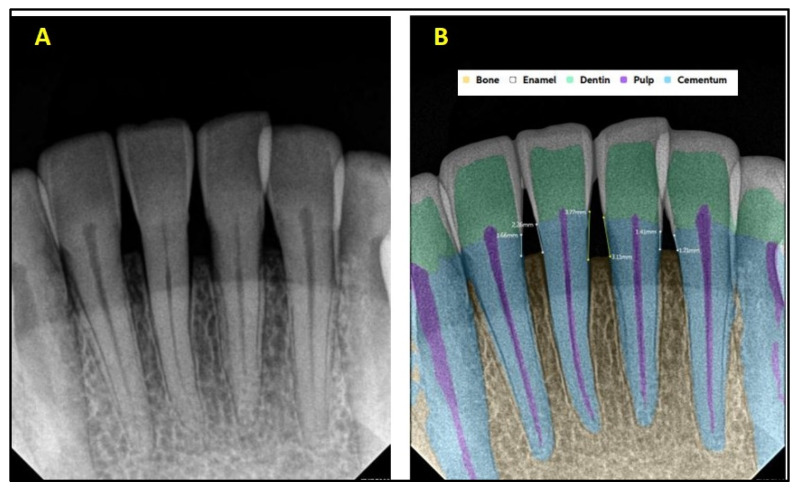
(**A**) Intraoral periapical radiographic image of mandibular anterior teeth; (**B**) analysis made by artificial intelligence software depicting bone loss.

Figure 1 explains the methodology used in the study. All the selected 300 IOPARs were fed into the AI model for detection of the predefined features. There was no collaboration between the researchers and the AI manufacturers during this research. The reports generated by the AI model were compared with the reference data to evaluate the diagnostic accuracy of the AI model (Figure 2, Figure 3, Figure 4, Figure 5, Figure 6, Figure 7, Figure 8 and Figure 9). In the second phase of the study, thirty dental interns and thirty dental specialists were randomly selected. Each participant was randomly assigned five IOPARs and was asked to detect and diagnose the predefined conditions. All the X-rays were blinded to examiners. Subsequently, all the participants were requested to reassess the IOPARs, this time with the assistance of the AI model. A washout period of 1 week was kept between the two reading sessions. All the data was recorded using a self-designed Performa.

### 2.3. Statistical Analysis

Epi info version 7.2. was used for data analysis. Frequency and percentage were used to describe the categorical variables. A chi-square test of a 2 × 2 contingency table was used to determine if there was a statistically significant difference between groups.

This evaluation utilized diagnostic metrics, such as sensitivity, specificity, positive predictive value (PPV), negative predictive value (NPV), accuracy, Youden’s index, diagnostic odds ratio (DOR), and likelihood ratios (LR+ and LR−). Sensitivity measures the proportion of true positive cases correctly identified by the model. It is calculated as TP/(TP + FN) × 100. Specificity measures the proportion of true negative cases correctly identified. It is calculated as TN/(TN + FP) × 100. PPV represents the probability that a positive test result is truly positive. It is calculated as TP/(TP + FP) × 100. NPV represents the probability that a negative test result is truly negative. It is calculated as TN/(TN + FN) × 100. Accuracy is a metric that evaluates the overall proportion of correct classifications. It is calculated as (TP + TN)/(TP + TN + FP + FN) × 100. Youden’s index measures the accuracy of a diagnostic test. It is calculated as (sensitivity + specificity) − 1 (Table 1). A *p* value < 0.05 was considered as a cutoff point for being statistically significant.

## 3. Results

A total of 1030 teeth from three hundred periapical radiographs were included in this evaluation. The periapical radiographs were evaluated by radiologists (reference data) and AI software, interns with and without the help of AI software, and specialists with and without the help of AI software. Each stage of the evaluation assessed eight dental conditions: caries, PA lesions, crowns, open crown margins, restoration, endodontic treatment, calculus, and marginal bone loss.

Table 2 shows the diagnostic metrics for the results of analysis by radiologists and AI software. The AI software correctly identified 721 out of 792 cases of caries (sensitivity: 91.0%), with a high PPV of 95.9%. It correctly ruled out caries in 207 out of 238 non-caries cases (specificity: 87.0%), with an NPV of 74.5%. The difference between the AI software and radiologist diagnoses of dental caries was statistically significant (*p* value < 0.0001).

Moreover, the AI software correctly identified 233 out of 269 cases with PA lesions (sensitivity: 86.6%), with a high PPV of 94.7%. It correctly ruled out cases with PA lesions in 748 out of 761 cases without PA lesions (specificity: 98.3%), with an NPV of 95.4%. The difference between the AI software and radiologist diagnoses of PA lesions was statistically significant (*p* value < 0.0001).

The sensitivity of the AI software in detecting crowns, open crown margins, restoration, endodontic treatment, calculus, and marginal bone loss was 97.1%, 82.6%, 89.3%, 93.4%, 80.2%, and 91.1%, respectively. The specificity of the AI software in detecting crowns, open crown margins, restoration, endodontic treatment, calculus, and marginal bone loss was 99.6%, 91.9%, 96.4%, 99.3%, 97.8%, and 93.1%, respectively. The differences between the AI software and radiologist diagnoses of crowns, open crown margins, restoration, endodontic treatment, calculus, and marginal bone loss were statistically significant (all *p* values < 0.0001).

**Figure 10:**
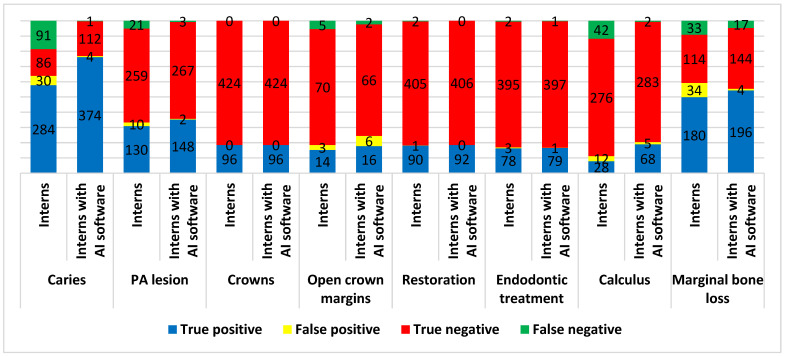
The diagnosis results of different conditions by dental interns with and without AI software.

Figure 10 compares the number of true and false positive and negative diagnoses of caries, PA lesions, crowns, open crown margins, restoration, endodontic treatment, calculus, and marginal bone loss identified by interns with and without the help of AI software.

Table 3 compares the diagnostic metrics between interns with and without the help of AI software for various conditions. The results showed that interns using AI software demonstrated higher accuracy, sensitivity, specificity, PPV, NPV, and Youden’s index in the detection of caries, PA lesions, restoration, endodontic treatment, calculus, and marginal bone loss compared to interns without AI software. However, these differences were only statistically significant in accuracy and sensitivity for caries detection (difference = 23.62% and 24.00%, respectively, and a *p* value = 0.0037 and 0.0103, respectively) and sensitivity for calculus detection (difference = 57.14% and *p* value = 0.0014).

For crown detection, interns with and without AI software achieved perfect diagnosis, with 100% accuracy, sensitivity, specificity, PPV, NPV, and a Youden’s index of 1.000 (*p* values of 1.0000 for accuracy, sensitivity, specificity). Conversely, in detecting open crown margins, the intern without the help of AI software displayed higher accuracy, specificity, and PPV compared to the intern with the help of AI software.

**Figure 11:**
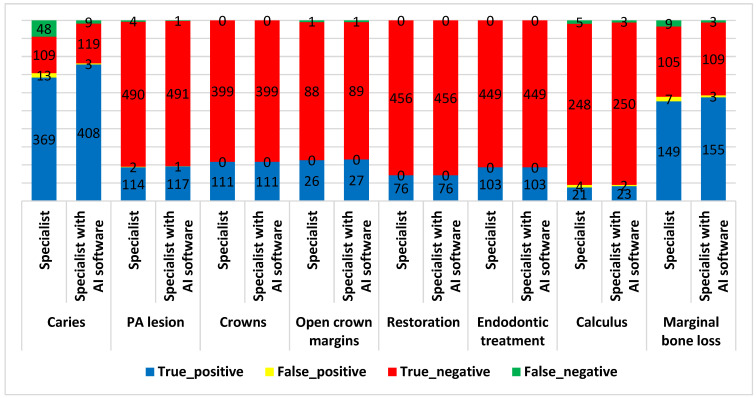
The diagnosis results of different conditions by specialists with and without AI software.

Figure 11 compares the number of true and false positive and negative diagnoses of caries, PA lesions, crowns, open crown margins, restoration, endodontic treatment, calculus, and marginal bone loss identified by specialists with and without the help of AI software.

Table 4 shows the diagnostic metrics between specialists with and without the help of AI software for different conditions. The results showed that the specialists using AI software achieved slightly higher accuracy, sensitivity, specificity, PPV, NPV, and Youden’s index in detecting caries, PA lesions, calculus, and marginal bone loss compared to those not using AI software.

The highest difference between specialists with and without AI software was observed in the accuracy, sensitivity, and specificity of caries detection (9.09%, 9.35%, and 8.20%, respectively). However, these differences were not statistically significant (*p* value > 0.05). Both specialists, with and without the assistance of AI software, demonstrated perfect accuracy in detecting crowns, open crown margins, restorations, and endodontic treatments. They achieved 100% accuracy, sensitivity, specificity, positive predictive value (PPV), negative predictive value (NPV), and a Youden’s index of 1.000. These results indicate that AI software did not provide additional benefits in these areas.

**Figure 12:**
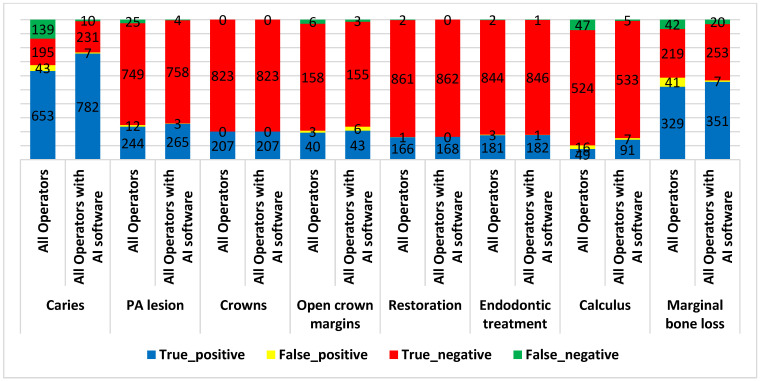
The diagnosis results of different conditions by operators with and without AI software.

Figure 12 compares the number of true and false positive and negative diagnoses of caries, PA lesions, crowns, open crown margins, restoration, endodontic treatment, calculus, and marginal bone loss identified by all operators with and without the help of AI software.

The results showed that operators (interns and specialists) using AI software revealed higher accuracy, sensitivity, specificity, PPV, NPV, and Youden’s index in the detection of caries, PA lesions, restoration, endodontic treatment, calculus, and marginal bone loss compared to those not using AI software (Table 5). However, these differences were only statistically significant in accuracy and sensitivity for caries detection (difference = 16.02% and 16.29%, respectively, and a *p* value = 0.0055 and 0.0136, respectively) and sensitivity for calculus detection (difference = 43.75% and *p* value = 0.0065).

Operators (interns and specialists) who either utilized or did not utilize AI software achieved perfect crown detection, with 100% accuracy, sensitivity, specificity, PPV, NPV, and a Youden’s index of 1.000 (*p* values of 1.0000 for accuracy, sensitivity, specificity).

## 4. Discussion

The use of AI in healthcare represents one of the most significant advancements in recent times. With continuous technological upgrades, these AI-based models promise safer and more predictable treatment options in the future. However, the application of AI in dentistry has not been explored to the same extent. The available literature assessing the diagnostic capabilities of this software is limited and requires more robust data. The present study aimed to evaluate the diagnostic accuracy of AI software using periapical radiographs and to compare the results with assessments made by dentists of varying experience levels. The findings indicated that the AI software demonstrated high sensitivity and specificity in detecting the conditions tested; therefore, the first null hypothesis was rejected. However, there was a noticeable difference in the accuracy of detecting various conditions. Furthermore, the study found that the use of AI software enhanced the overall diagnostic accuracy of dentists when identifying most of the tested conditions, though significant differences were only observed in the detection of caries and calculus. This variability in detection accuracy led to the partial rejection of the second null hypothesis.

### 4.1. AI in the Detection of Caries

For the long-term maintenance of teeth, accurately diagnosing carious lesions at an early stage is essential. Since the management of these lesions involves irreversible treatment procedures, the specificity of the diagnostic tool—meaning its ability to correctly identify true negatives—is more critical than its sensitivity, or its ability to identify true positives. In the present study, AI software demonstrated high specificity (87.0%) and high sensitivity (91.0%). There were statistically significant differences between the AI results and the reference data. These findings are consistent with those of Lee et al. [36], who reported high specificity (83%) and sensitivity (81%) of a tested Deep CNN in the detection of carious lesions. Similarly, Moran et al. [37] tested various CNN models (Inception and ResNet) and found specificity ranging from 80% to 100% for detecting both incipient and advanced carious lesions. The ability of a dentist to detect caries depends on their perceptual skills, expertise, and experience. In our study, the use of AI software significantly improved the dentists’ diagnostic capabilities for identifying carious lesions. The specificity for caries detection among all operators (interns and specialists) was reported at 97.06% with AI software and 81.93% without it. Correspondingly, the sensitivity values were 98.74% with AI and 82.45% without it. This indicates a statistically significant improvement in both specificity and sensitivity when AI software was employed. There were also variations in performance between interns and specialists, likely due to differences in experience and training levels. Interns experienced a greater improvement in specificity (from 74.14% to 96.55%) and sensitivity (from 75.73% to 99.73%) with the use of AI. In contrast, dental specialists showed a smaller increase, with specificity rising from 89.34% to 97.54% and sensitivity from 88.49% to 97.84% when using AI. Overall accuracy in caries detection improved from 88.68% to 97.77% for all operators using the AI software. The results of this study partially align with those of Merten et al. [11], who reported an increase in accuracy (from 93% to 94%) and sensitivity (from 72% to 81%) but no change in specificity when AI software was utilized for caries diagnosis. The discrepancies may be attributed to differences in AI software used and varying levels of experience and training among the participating dentists.

### 4.2. AI in the Detection of Bone Loss

A precise radiographic evaluation of alveolar bone levels is essential for diagnosing periodontal disease. By assessing the extent of bone loss, a dentist can classify the disease’s stage and plan appropriate treatment. However, accurately interpreting bone loss on radiographs is multifactorial, which can influence both diagnosis and treatment planning. In the present study, AI software demonstrated high sensitivity (91.1%) and specificity (93.1%) in detecting alveolar bone loss, and there were statistically significant differences between the AI results and reference data. These findings align with studies by Kim et al. [27] and Krois et al. [28], which also reported high accuracy of AI software in detecting alveolar bone loss. However, direct comparisons are challenging because those studies employed panoramic radiographs, while our study utilized intraoral periapical radiographs. The introduction of AI software enhanced operators’ diagnostic capabilities, although the differences were not statistically significant. Overall, the accuracy of detecting alveolar bone loss increased from 86.85% to 95.72% for all operators using AI software. This is consistent with findings by Chen et al. [29], who reported an AI detection accuracy of 97%, compared to 76–78% accuracy achieved by dentists without AI assistance. Similarly, Lee et al. [38] indicated that the use of AI led to a higher detection accuracy of 90% for alveolar bone loss compared to the 76–78% accuracy of dental clinicians.

### 4.3. AI in the Detection of Periapical Lesions

Apical periodontitis is an inflammatory lesion that can be seen radiographically around the apex of the tooth root in response to pulpal necrosis or periodontitis. Early detection of these lesions can significantly improve the success rate of treatment. The present study reported a high sensitivity of 86.6% and a specificity of 98.3% for the tested AI software, with the differences being statistically significant. These results align with findings from Issa et al. [30], who reported a sensitivity of 92.3% and specificity of 97.87%. A similar study by İçöz D et al. [31] noted a high precision of 0.56 for AI software in detecting roots affected by apical periodontitis. They also found that precision was greater for detecting apical periodontitis in the mandibular jaw compared to the maxillary jaw. This difference may be due to overlapping structures and lower contrast between the lesions and anatomical features in panoramic radiographs of the maxillary arch. The accuracy of detecting periapical lesions improved from 96.41% to 99.32% with the use of AI software by all operators. However, there were variations in improvement levels; interns showed an increase from 92.62% to 98.81%, while specialists had minimal improvements, ranging from 99.02% to 99.67%.

### 4.4. AI in Detecting Open Crown Margins and Calculus

The AI software reported sensitivity and specificity values of 82.6% and 91.9%, respectively, for detecting open crown margins, and 80.2% and 97.8% for detecting calculus. Identifying open crown margins is crucial, as early detection and intervention can prevent damage to the abutment tooth caused by microleakage. Likewise, the detection and removal of calculus are essential for preventing the loss of supporting structures. The use of AI software by dental operators showed no significant difference in detecting open crown margins. However, there was an improvement in the detection of calculus, which increased from 90.09% to 98.11%, with variations noted between interns and specialists.

### 4.5. AI in the Detection of Dental Treatments (Crowns, Restorations, and Endodontic Treatments)

The AI software reported sensitivity values of 97.1% for crowns, 89.3% for restorations, and 93.4% for endodontic treatments. The corresponding specificity values were 99.6% for crowns, 96.4% for restorations, and 99.3% for endodontic treatments. These results align with the findings by Bonfanti-Gris et al. [21], who also reported high sensitivity and specificity for the AI software. However, a direct comparison between the studies is not possible due to differences in the AI software used and the types of radiographic images evaluated; the current study utilized intraoral periapical radiographs, while Bonfanti-Gris et al. employed panoramic radiographs. The use of AI software by specialists did not provide any additional advantage in detecting crowns, restorations, and endodontic treatments, as they achieved perfect accuracy both with and without AI. In contrast, dental interns showed improved accuracy with the use of AI software for detecting endodontic treatments (increasing from 98.95% to 99.58%) and restorations (from 99.40% to 100%). However, there was no additional benefit in detecting crowns, as interns also demonstrated perfect accuracy without the AI software. Although these dental treatments can be detected easily without AI, the software can still be valuable for educating and discussing options with patients during the treatment planning phase.

In the present study, the AI software tested demonstrated high accuracy, sensitivity, and specificity in identifying a range of periodontal and restorative conditions. The study has several strengths and limitations. Among the strengths is a large and balanced dataset that included all the tested conditions, a robust methodology, and the use of previously validated AI software. This software highlights the identified conditions in different colors, facilitating easy identification by dentists. Furthermore, the study focused on a commonly encountered set of conditions that can assist dentists in diagnosis, treatment planning, and patient education. However, there are several limitations to the study. First, it relied solely on two-dimensional periapical radiographs to create the reference dataset. Incorporating additional clinical diagnostic methods and three-dimensional radiographs could have enhanced the findings. Second, all the radiographs were of high quality, which may have inflated the diagnostic accuracy of the AI software. The performance of the AI tool may be affected when used to evaluate low-quality heterogeneous images. The clinicians should ensure that the images taken in their clinical setting are of high quality using standardized recommended techniques for acquiring the X-ray. Additionally, the images should be monitored and checked for quality before being evaluated by the AI tool to minimize errors. Also, to improve the generalizability of the software, techniques like data augmentation and adaptive thresholding should be used. Third, the results of this study cannot be generalized because all the images were obtained from three machines in a single dental clinic. Lastly, the study was restricted to permanent teeth, excluding deciduous teeth.

## 5. Conclusions

Within the limitations of the study, it can be concluded that the tested AI software has high accuracy in detecting the tested periodontal and restorative dental conditions in IOPARs. The use of AI software enhanced the diagnostic capabilities of dental operators. The present study used AI software to detect a clinically useful set of conditions, which can help dental operators in fast and accurate diagnosis and provide high-quality treatment to their patients. Future research that employs advanced AI software capable of evaluating a broader range of clinical conditions could be beneficial for dental practitioners.

## Figures and Tables

**Table 1:** Criteria for diagnosis by the radiologist.

		Present	Absent	Total
AI software	Positive	TP	FP	TP + FP
	Negative	FN	TN	FN + TN
	Total	TP + FP	FP + TN	TP + FP + FP + TN

TP: true positive; FP: false positive; FN: false negative; TN: true negative.

**Table 2:** The diagnostic tests and metrics for reference data and AI software for different conditions.

Conditions	AI Software	Diagnosis by Radiologist			Sensitivity	Specificity	PPV	NPV	*p* Value
		Present	Absent	Total					
Caries	Positive	721	31	752	91.0%	87.0%	95.9%	74.5%	<0.0001
	Negative	71	207	378					
	Total	792	238	1030					
PA lesions	Positive	233	13	246	86.6%	98.3%	94.7%	95.4%	<0.0001
	Negative	36	748	784					
	Total	269	761	1030					
Crowns	Positive	201	3	204	97.1%	99.6%	98.5%	99.3%	<0.0001
	Negative	6	820	826					
	Total	207	823	1030					
Open crown margins	Positive	38	13	51	82.6%	91.9%	74.5%	94.9%	<0.0001
	Negative	8	148	156					
	Total	46	161	207					
Restoration	Positive	150	31	181	89.3%	96.4%	82.9%	97.9%	<0.0001
	Negative	18	831	849					
	Total	168	862	1030					
Endodontic treatment	Positive	171	6	177	93.4%	99.3%	96.6%	98.6%	<0.0001
	Negative	12	841	853					
	Total	183	847	1030					
Calculus	Positive	77	12	89	80.2%	97.8%	86.5%	96.5%	<0.0001
	Negative	19	528	547					
	Total	96	540	636					
Marginal bone loss	Positive	338	18	356	91.1%	93.1%	94.9%	88.0%	<0.0001
	Negative	33	242	275					
	Total	371	260	631					

AI: artificial intelligence; PPV: positive predictive value; NPV: negative predictive value; PA: periapical. *p* value from a chi-square test of a 2 × 2 contingency table to compare between groups.

**Table 3:** Comparing the diagnostic metrics between interns with and without AI software for different conditions.

Conditions	Operators	Accuracy	Sensitivity	Specificity	PPV	NPV	Youden’s Index
Caries	Interns	75.36%	75.73%	74.14%	90.45%	48.59%	0.499
	Interns with AI software	98.98%	99.73%	96.55%	98.94%	99.12%	0.963
	Difference	23.62%	24.00%	22.41%			
	*p* value	0.0037	0.0103	0.1739			
PA lesions	Interns	92.62%	86.09%	96.28%	92.86%	92.50%	0.824
	Interns with AI software	98.81%	98.01%	99.26%	98.67%	98.89%	0.973
	Difference	6.19%	11.92%	2.98%			
	*p* value	0.5121	0.4358	0.8041			
Crowns	Interns	100.00%	100.00%	100.00%	100.00%	100.00%	1.000
	Interns with AI software	100.00%	100.00%	100.00%	100.00%	100.00%	1.000
	Difference	0.00%	0.00%	0.00%			
	*p* value	1.0000	1.0000	1.0000			
Open crown margins	Interns	91.30%	73.68%	95.89%	82.35%	93.33%	0.696
	Interns with AI software	91.11%	88.89%	91.67%	72.73%	97.06%	0.806
	Difference	−0.19%	15.21%	−4.22%			
	*p* value	0.9463	0.7029	0.8504			
Restoration	Interns	99.40%	97.83%	99.75%	98.90%	99.51%	0.976
	Interns with AI software	100.00%	100.00%	100.00%	100.00%	100.00%	1.000
	Difference	0.60%	2.17%	0.25%			
	*p* value	0.9921	0.9163	0.9802			
Endodontic treatment	Interns	98.95%	97.50%	99.25%	96.30%	99.50%	0.967
	Interns with AI software	99.58%	98.75%	99.75%	98.75%	99.75%	0.985
	Difference	0.63%	1.25%	0.50%			
	*p* value	0.9450	0.9548	0.9599			
Calculus	Interns	84.92%	40.00%	95.83%	70.00%	86.79%	0.358
	Interns with AI software	98.04%	97.14%	98.26%	93.15%	99.30%	0.954
	Difference	13.12%	57.14%	2.43%			
	*p* value	0.1842	0.0014	0.8330			
Marginal bone loss	Interns	81.44%	84.51%	77.03%	84.11%	77.55%	0.615
	Interns with AI software	94.18%	92.02%	97.30%	98.00%	89.44%	0.893
	Difference	12.74%	7.51%	20.27%			
	*p* value	0.1823	0.5475	0.1716			

AI: artificial intelligence; PPV: positive predictive value; NPV: negative predictive value; PA: periapical. *p* value from a chi-square test of a 2 × 2 contingency table to compare the accuracy and sensitivity between two groups.

**Table 4:** Comparing the diagnostic metrics between specialists with and without AI software for different conditions.

Conditions	Operators	Accuracy	Sensitivity	Specificity	PPV	NPV	Youden’s Index
Caries	Specialists	88.68%	88.49%	89.34%	96.60%	69.43%	0.778
	Specialists with AI software	97.77%	97.84%	97.54%	99.27%	92.97%	0.954
	Difference	9.09%	9.35%	8.20%			
	*p* value	0.2660	0.3140	0.6339			
PA lesions	Specialists	99.02%	96.61%	99.59%	98.28%	99.19%	0.962
	Specialists with AI software	99.67%	99.15%	99.80%	99.15%	99.80%	0.989
	Difference	0.65%	2.54%	0.21%			
	*p* value	0.9351	0.8884	0.9820			
Crowns	Specialists	100.00%	100.00%	100.00%	100.00%	100.00%	1.000
	Specialists with AI software	100.00%	100.00%	100.00%	100.00%	100.00%	1.000
	Difference	0.00%	0.00%	0.00%			
	*p* value	1.0000	1.0000	1.0000			
Open crown margins	Specialists	99.13%	96.30%	100.00%	100.00%	98.88%	0.963
	Specialists with AI software	99.15%	96.43%	100.00%	100.00%	98.89%	0.964
	Difference	0.02%	0.13%	0.00%			
	*p* value	0.9626	0.9972	1.0000			
Restoration	Specialists	100.00%	100.00%	100.00%	100.00%	100.00%	1.000
	Specialists with AI software	100.00%	100.00%	100.00%	100.00%	100.00%	1.000
	Difference	0.00%	0.00%	0.00%			
	*p* value	1.0000	1.0000	1.0000			
Endodontic treatment	Specialists	100.00%	100.00%	100.00%	100.00%	100.00%	1.000
	Specialists with AI software	100.00%	100.00%	100.00%	100.00%	100.00%	1.000
	Difference	0.00%	0.00%	0.00%			
	*p* value	1.0000	1.0000	1.0000			
Calculus	Specialists	96.76%	80.77%	98.41%	84.00%	98.02%	0.792
	Specialists with AI software	98.20%	88.46%	99.21%	92.00%	98.81%	0.877
	Difference	1.44%	7.69%	0.80%			
	*p* value	0.9027	0.8245	0.9493			
Marginal bone loss	Specialists	94.07%	94.30%	93.75%	95.51%	92.11%	0.881
	Specialists with AI software	97.78%	98.10%	97.32%	98.10%	97.32%	0.954
	Difference	3.71%	3.80%	3.57%			
	*p* value	0.7536	0.8059	0.8450			

AI: artificial intelligence; PPV: positive predictive value; NPV: negative predictive value; PA: periapical. *p* value from a chi-square test of a 2 × 2 contingency table to compare the accuracy and sensitivity between two groups.

**Table 5:** Comparing the diagnostic metrics between all operators (interns and specialists) with and without AI software for different conditions.

Conditions	Operators (Interns and Specialists)	Accuracy	Sensitivity	Specificity	PPV	NPV	Youden’s Index
Caries	Interns and specialists	82.33%	82.45%	81.93%	93.82%	58.38%	0.644
	Interns and specialists with AI software	98.35%	98.74%	97.06%	99.11%	95.85%	0.958
	Difference	16.02%	16.29%	15.13%			
	*p* value	0.0055	0.0136	0.2048			
PA lesions	Interns and specialists	96.41%	90.71%	98.42%	95.31%	96.77%	0.891
	Interns and specialists with AI software	99.32%	98.51%	99.61%	98.88%	99.48%	0.981
	Difference	2.91%	7.80%	1.19%			
	*p* value	0.6348	0.5045	0.8695			
Crowns	Interns and specialists	100.00%	100.00%	100.00%	100.00%	100.00%	1.000
	Interns and specialist with AI software	100.00%	100.00%	100.00%	100.00%	100.00%	1.000
	Difference	0.00%	0.00%	0.00%			
	*p* value	1.0000	1.0000	1.0000			
Open crown margins	Interns and specialists	95.65%	86.96%	98.14%	93.02%	96.34%	0.851
	Interns and specialists with AI software	95.65%	93.48%	96.27%	87.76%	98.10%	0.898
	Difference	0.00%	6.52%	−1.87%			
	*p* value	1.0000	0.8113	0.9039			
Restoration	Interns and specialists	99.71%	98.81%	99.88%	99.40%	99.77%	0.987
	Interns and specialists with AI software	100.00%	100.00%	100.00%	100.00%	100.00%	1.000
	Difference	0.29%	1.19%	0.12%			
	*p* value	0.9627	0.9382	0.9864			
Endodontic treatment	Interns and specialists	99.51%	98.91%	99.65%	98.37%	99.76%	0.986
	Interns and specialists with AI software	99.81%	99.45%	99.88%	99.45%	99.88%	0.993
	Difference	0.30%	0.54%	0.23%			
	*p* value	0.9626	0.9703	0.9725			
Calculus	Interns and specialists	90.09%	51.04%	97.04%	75.38%	91.77%	0.481
	Interns and specialists with AI software	98.11%	94.79%	98.70%	92.86%	99.07%	0.935
	Difference	8.02%	43.75%	1.66%			
	*p* value	0.2899	0.0065	0.8440			
Marginal bone loss	Interns and specialists	86.85%	88.68%	84.23%	88.92%	83.91%	0.729
	Interns and specialists with AI software	95.72%	94.61%	97.31%	98.04%	92.67%	0.919
	Difference	8.87%	5.93%	13.08%			
	*p* value	0.2328	0.5422	0.2569			

AI: artificial intelligence; PPV: positive predictive value; NPV: negative predictive value; PA: periapical. *p* value from a chi-square test of a 2 × 2 contingency table to compare the accuracy and sensitivity between two groups.

## Data Availability

The data that support the findings of this study are available from the corresponding author upon reasonable request.
